# Adult patients with severe respiratory syncytial virus infections in the emergency department

**DOI:** 10.1097/MD.0000000000039265

**Published:** 2024-09-27

**Authors:** David Fistera, Christian M. Kramer, Randi Katrin Manegold, Carina Elsner, Ulf Dittmer, Christian Taube, Clemens Kill, Joachim Risse

**Affiliations:** a Center of Emergency Medicine, University Hospital Essen, Essen, Germany; b Institute for Virology, University Hospital Essen, University of Duisburg-Essen, Essen, Germany; c Department of Pulmonary Medicine, University Medicine Essen – Ruhrlandklinik, Essen, Germany.

**Keywords:** adult, comorbidities, emergency department, point-of-care testing PCR, respiratory syncytial virus, RSV

## Abstract

Respiratory syncytial virus (RSV) is a seasonal virus known to cause significant morbidity in pediatric patients; however, morbidity in adult patients has not been well investigated. We aimed to characterize adult patients with RSV infection in the emergency department (ED) and their clinical course. During the winter term 2022/23, all adult ED patients were screened for RSV, severe acute respiratory syndrome coronavirus type 2, and influenza infection using point-of-care polymerase chain reaction tests. All symptomatic RSV+ patients were further characterized based on their clinical presentation and course. A group comparison between RSV+ inpatients and RSV+ outpatients was conducted. The potential risk factors for inpatient treatment were evaluated using univariate and multivariate analyses. Of the 135 symptomatic RSV+ patients, 51.9% (70/135) were inpatients. Their length of stay were 9.4 (±10.4) days. Inpatients had a significantly higher mean age, lower oxygen saturation, higher leukocyte count, and higher C-reactive protein levels than outpatients. Among the preconditions, pulmonary diseases, tumors, and immunosuppression were significantly more frequent in the inpatient group. Thirty percent (21/70) of the inpatients required ICU treatment, 11% (8/70) required mechanical ventilation, and 9% (6/70) died. Malaise (*P* = .021, odds ratio 8.390) and detection of pulmonary infiltrations (*P* < .001, odds ratio 12.563) were the only independent predictors of inpatient treatment in the multivariate analysis. Our data show that RSV is a medically relevant pathogen among adult ED patients, often requiring inpatient treatment. In particular, elderly patients with some medical preconditions seem to be more prone to a severe course of infection requiring inpatient treatment. Lower respiratory tract involvement, proven by pulmonary infiltrates, seems to be crucial for a more severe disease course.

## 1. Introduction

Respiratory syncytial virus (RSV) is a negative-sense, single-stranded RNA virus that belongs to the *Pneumoviridae* family and is transmitted through respiratory droplets.^[1]^ It causes seasonal infection outbreaks during winter in the Northern Hemisphere and is well known for causing acute respiratory infectious diseases among younger children and infants.^[2]^ Due to the high attack rate, almost all children are affected by 2 years of age with a significant hospitalization rate and an estimated 127,700 worldwide deaths per year among children <5 years, especially in low- and middle-income countries.^[3,4]^

RSV infections also affect adults and are increasingly being recognized as a common cause of respiratory illness during the winter season.^[5]^ The clinical presentation varies from mild symptoms limited to the upper respiratory tract to severe lower respiratory tract infections and exacerbation of other diseases.^[6]^ Main risk factors for a prolonged and more severe course of RSV infection in adults include age, chronic cardiopulmonary diseases, and immunosuppression.^[7,8]^ A prospective study from 2017 to 2020 showed that the annual RSV incidence in hospitalized adults ranged from 44.2 to 58.9/10.000 with higher rates among older patients and those with cardiac conditions.^[9]^ Possible complications include respiratory failure with intensive care unit (ICU) administration and the need for mechanical ventilation.^[10]^ Patients with bone marrow transplants seem to have the highest mortality rate among RSV-infected individuals.^[11]^

These few studies provide the first hint that RSV is an important pathogen in adults, but RSV is not regularly tested or often not taken into consideration for a differential diagnosis; therefore, data concerning RSV in the adult population are limited. The coronavirus disease pandemic, with its challenges for the healthcare system, offered the opportunity to intensify polymerase chain reaction (PCR) testing for respiratory viruses in hospitals, which allows the determination of the prevalence of RSV infections at the time of presentation in the hospital. In addition, follow-up of inpatients can inform about the morbidity of RSV infections in adult patients.

This study aimed to investigate adult patients with symptomatic RSV infections in the emergency department (ED) of a German university hospital and their clinical course to evaluate the potential risk factors for inpatient treatment.

## 2. Methods

### 2.1. Patients

Between October 01, 2022 and March 31, 2023, all adult patients presenting to our tertiary care ED underwent point-of-care PCR testing for RSV, severe acute respiratory syndrome coronavirus type 2 (SARS-CoV-2), and influenza.

RSV positive (+) symptomatic patients were included when at least one of the following symptoms was present upon arrival: malaise, dyspnea, cough, fever, headache, nausea, chest pain, sore throat, and myalgia.

Inclusion criteria were:

- Age > 18 years presenting to the non-trauma emergency department.
- RSV+ in point-of-care testing PCR.
- Symptomatic at presentation (at least 1 clinical symptom of the above mentioned).

The study was approved by the institutional ethics committee “Ethik-Kommission der Medizinischen Fakultät der Universität Duisburg-Essen” (approval no. 23–11276-BO, 19/06/2023), and written patient consent was waived by the ethics committee because of the anonymized retrospective data.

This study was registered in the German trial registry (no. DRKS00032949).

### 2.2. Methods

Nasopharyngeal swabs were collected by trained nursing staff and analyzed using the *Xpert Xpress CoV-2/Flu/RSV plus* test on the *Cepheid Xpert Xpress* PCR system. All PCR results were electronically transferred to the Institute for Virology Essen and were technically and medically validated.

Data of all RSV+ patients were obtained from electronic medical records (ERPath, eHealth-Tec Innovations GmbH, Berlin, Germany; Medico, Cerner Health Services GmbH, Idstein, Germany). Missing data that could not be extracted from the patients’ records were excluded from statistical analysis. All patient data were fully anonymized before further analyses were performed.

Patient data were then further analyzed concerning vital parameters, laboratory values, medical preconditions, and further diagnosis and treatment.

Subgroups of in- and outpatients were compared for baseline data.

### 2.3. Statistical analyses

Groups of RSV+ inpatients and outpatients were compared using the *t* test for non-categorical data and Fisher Exact test for categorical data. The *t* test was used to evaluate the metric data. To assess equality of variance, the data were tested using Levene test. Welch *t* test was used to analyze metric data in the case of unequal variances. Results are reported as the mean ± standard deviation for metric variables. Fisher Exact test was used to compare categorical data. Categorical variables are expressed as percentages and odds ratios (OR) with 95% confidence intervals (95% CI). For both tests, statistical significance was defined as two-tailed *P* < .05. Adjustment for multiple testing was omitted because of the exploratory nature of the analyses.

Regarding the endpoint of inpatient admission, multivariate binary logistic regression analysis was performed, and meaningful parameters identified as significant (*P* < .05) in the univariate analysis were additionally examined through multivariate analysis. Variables for the multivariate logistic regression analysis were age with “age group > 60 years,” symptoms like “Malaise,” “Dyspnea,” “Fever,” “Headache,” the Oxygen saturation < 95%, lab parameter C-reactive protein > 7.0 mg/dL, pulmonary infiltrate and preexisting conditions like “Pulmonary” “Tumor” “Immunosuppression,” and “Hypertension.” As a measure of the explained variation, Nagelkerke *R*^2^ was used.

All data were processed using Excel software (Version 2017, Microsoft Corporation, Redmond, WA) and IBM SPSS Statistics (Version 27.0, IBM Corporation, Armonk, NY).

The complete anonymized dataset was uploaded as a separate file (Data S1, Supplemental Digital Content, RSV-data_file_final.xlsx, http://links.lww.com/MD/N593).

## 3. Results

### 3.1. General patient group

**Figure 1.**
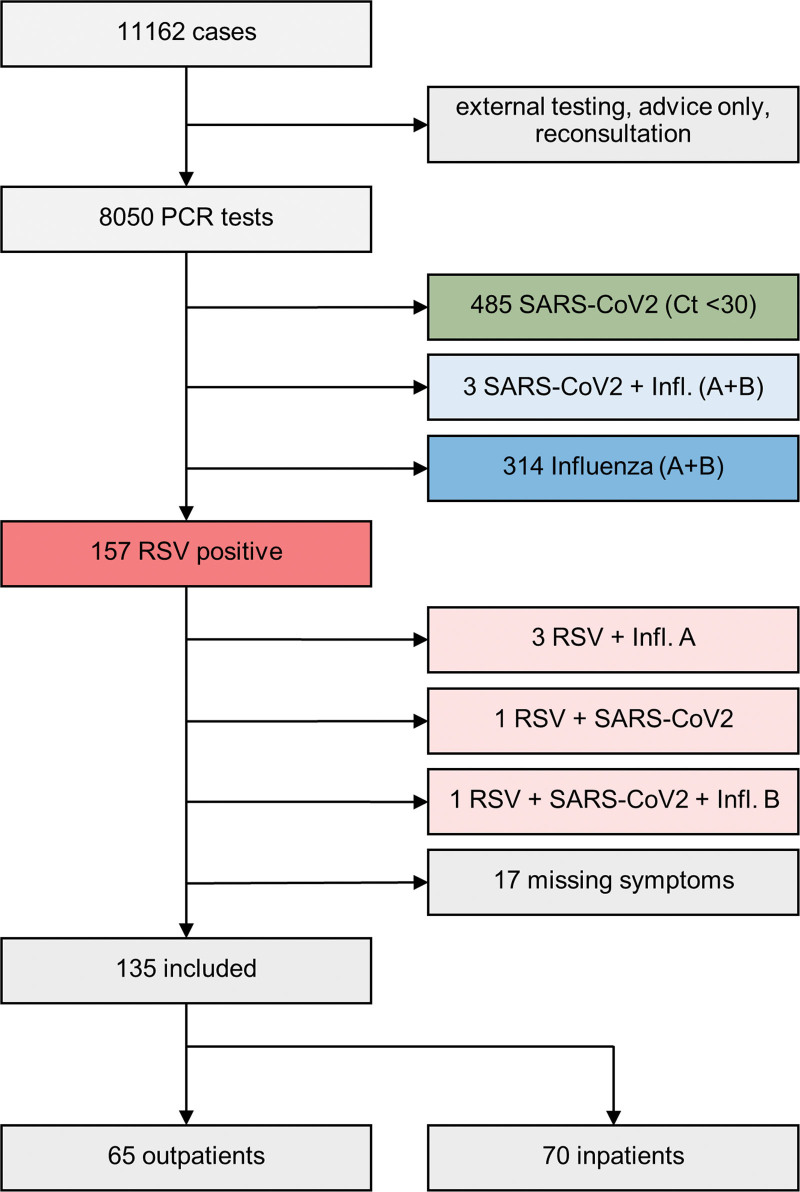
Study flow chart.

In the 6 months of the study, 8050 adult patients were tested for RSV, influenza, and SARS-CoV-2. Of these patients, 157 (2.0%) tested positive for RSV, 490 (6.1%) tested positive for SARS-CoV-2, and 321 (4.0%) tested positive for influenza A/B. Coinfections were rarely found, with 1 coinfection with RSV and SARS-CoV-2, 3 coinfections with RSV and influenza, and 1 coinfection with RSV, SARS-CoV-2, and influenza. In the following section, we focus our analysis on RSV infections only. Only 10.8% (17/157) of RSV+ patients presented without any defined respiratory symptoms (Fig. 1).

### 3.2. RSV patients in the ED

After applying our inclusion and exclusion criteria, 135 adult RSV patients were further analyzed with a mean age of 59 ± 20 years and male sex in 49% (66/135) of cases. The documented symptoms of the total 135 RSV+ patients were the following: 89% (120/135) reported malaise, 47% (64/135) dyspnea, 41% (56/135) cough, and 24% (33/135) fever as their major complaint upon presentation. The dataset and additional baseline data are presented in Table 1.

**Table 1:** Group comparison of RSV + outpatients versus inpatients: univariate analysis.

Category	All patients (n = 135) mean ± SD or n (%)	Outpatients (n = 65) mean ± SD or n (%)	Inpatients (n = 70) mean ± SD or n (%)	Fisher Exact test or *t* test *P*-value	Odds ratio (95 % CI)
Patient characteristics					
Age (years)	59 ± 20	53 ± 23	64 ± 15	**<.001**	
Age > 60 years	73 (54%)	27 (41%)	46 (66%)	**.006**	2.627 (1.305–5.288)
Male sex	66 (49%)	31 (48%)	35 (50%)	.864	0.912 (0.464–1.792)
Symptoms					
Malaise	120 (89%)	53 (82%)	67 (95%)	**.012**	5.057 (1.357–18.843)
Dyspnea	64 (47%)	22 (34%)	42 (60%)	**.003**	2.932 (1.453–5.915)
Cough	56 (41%)	25 (38%)	31 (48%)	.600	1.272 (0.640–2.528)
Fever	33 (24%)	10 (15%)	23 (33%)	**.027**	2.691 (1.164–6.224)
Headache	18 (13%)	13 (20%)	5 (7%)	**.041**	0.308 (0.103–0.919)
Nausea	18 (13%)	8 (12%)	10 (14%)	.804	1.188 (0.438–3.221)
Chest pain	16 (12%)	11 (17%)	5 (7%)	.110	0.378 (0.124–1.154)
Sore throat	15 (11%)	8 (12%)	7 (10%)	.786	0.792 (0.270–2.321)
Myalgia	11 (8%)	8 (1g2%)	3 (4%)	.118	0.319 (0.081–1.259)
Vital signs					
Heart rate (bpm)	96 ± 21	92 ± 19	99 ± 22	.409	
Systolic blood pressure (mm Hg)	132 ± 23	128 ± 23	135 ± 22	.681	
Diastolic blood pressure (mm Hg)	79 ± 15	78 ± 14	80 ± 16	.702	
Oxygen saturation (%)	95 ± 5	97 ± 3	93 ± 7	**<.001**	
Oxygen saturation < 95%	47 (35%)	14 (21%)	33 (47%)	**.002**	3.249 (1.527–6.912)
Respiratory rate (bpm)	21 ± 5	20 ± 4	22 ± 5	.625	
Temperature (°C)	36.8 ± 1.1	36.6 ± 0.8	37.0 ± 1.2	.089	
Lab results					
Leukocytes (/nL)	10.1 ± 6.3	9.4 ± 4.2	10.9 ± 7.6	**.043**	
Leukocytes (>10.0/nL)	50 (37%)	21 (32%)	29 (41 %)	.715	1.213 (0.591–2.487)
C-reactive protein (mg/dL)	6.8 ± 8.7	4.4 ± 5.8	8.8 ± 10.1	**<.001**	
C-reactive protein > 7.0 mg/dL	37 (27%)	11 (17%)	26 (37%)	**.031**	2.525 (1.116–5.710)
Procalcitonin ng/mL	2.2 ± 7.6	1.5 ± 6.9	2.5 ± 7.9	.370	
Ct value	27.3 ± 6.2	27.2 ± 6.5	27.4 ± 5.9	.307	
Imaging					
Chest X-ray	40 (30%)	15 (23%)	25 (36%)	.132	1.852 (0.869–3.945)
CT scan	37 (27%)	3 (5%)	34 (49%)	**<.001**	19.519 (5.593–68.116)
Pulmonary infiltrate	44 (33%)	5 (8%)	39 (56%)	**<.001**	15.097 (5.406–42.160)
Preexisting conditions					
Pulmonary	44 (33%)	14 (22%)	30 (43%)	**.010**	2.732 (1.281–5.827)
Cardiac	49 (36%)	20 (31%)	29 (41%)	.251	1.591 (0.783–3.236)
Renal	16 (12%)	4 (6%)	12 (17%)	.063	3.155 (0.962–10.343)
Hepatic	4 (3%)	2 (3%)	2 (3%)	1.000	0.926 (0.127–6.776)
Tumor	39 (29%)	12 (18%)	27 (39%)	**.013**	2.773 (1.259–6.111)
Transplantation	12 (9%)	3 (5%)	9 (13%)	.131	3.049 (0.788–11.804)
Immunosuppression	14 (10%)	3 (5%)	11 (16%)	**.047**	3.853 (1.024–14.502)
HIV	2 (1%)	2 (3%)	0 (0%)	.230	–
Hypertension	67 (50%)	21 (32%)	46 (66%)	**<.001**	4.016 (1.961–8.224)
Diabetes	23 (17%)	7 (11%)	16 (23%)	.070	2.455 (0.938–6.427)
Outcome (inpatients only)					
Length of stay (days)			9.4 ± 10.4		
IMC/ICU administration			21 (30%)		
NIV/high-flow			8 (11%)		
Intubation			8 (11%)		
Mortality			6 (9%)		

*P* < 0.05.

The potential risk factors for inpatient treatment were also evaluated. Documented medical preconditions of RSV+ patients were as follows: arterial hypertension in 50% (67/135), cardiac disease in 36% (49/135), pulmonary disease in 33% (44/135), and tumors in 29% (39/135). A total of 10% (14/135) were immunosuppressed (8 stem cell transplants, 3 solid organ transplants, 2 collagenoses, and 1 common variable immunodeficiency) (Table 1).

Of the 135 RSV+ patients, 51.8% (70/135) were prospective inpatients.

### 3.3. Group comparison of in- and outpatients

Among RSV+ patients, inpatients were significantly older than outpatients (mean age 64 ± 15 years vs 53 ± 23 years, *P* < .001) and showed significantly more preconditions (Table 1). Pulmonary diseases (30/70 vs 14/65, *P* = .01), tumors (27/70 vs 12/65, *P* = .013), and immunosuppression (16/70 vs 3/65, *P* = .047) were significantly more prevalent among inpatients.

**Figure 2.**
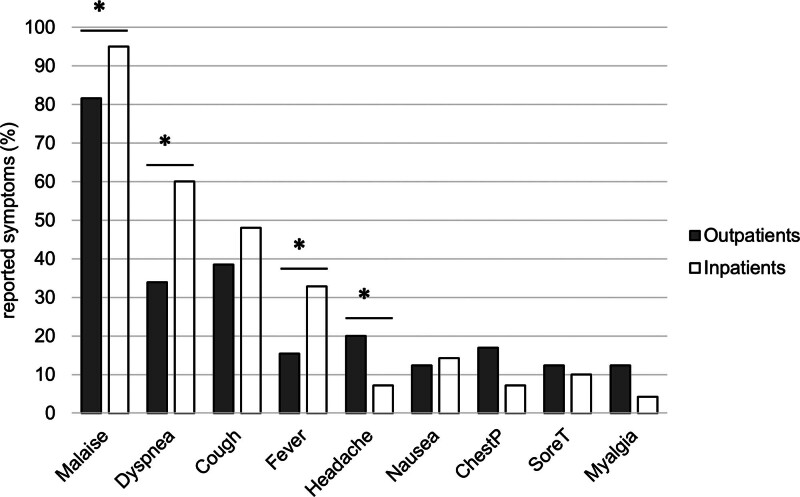
Reported symptoms of in- and outpatients.

An analysis of symptoms upon RSV infection revealed that malaise (OR 5.057, CI 1.357–18.843, *P* = .012), dyspnea (OR 2.932, CI 1.453–5.915, *P* = .003), and fever (OR 2.691, CI 1.164–6.224, *P* = .027) were significantly more often detected in inpatients than in outpatients, whereas headache was significantly less frequent in this group (OR 0.308, CI 0.103–0.919, *P* = .041) (Fig. 2). The onset of these symptoms was reported earlier in the inpatient group than in the outpatient group (4.5 ± 5.3 days vs 2.6 ± 2.8 days, *P* < .001).

To obtain a rough estimate of viral loads in RSV+ patients, we compared the PCR cycle thresholds between inpatients and outpatients. The cycle threshold in the nasopharyngeal swabs, however, were not significantly different between the groups (27.4 ± 5.9 vs 27.2 ± 6.5, *P* = .307).

In addition, inpatients presented with lower oxygen saturation upon arrival (93 ± 7% vs 97 ± 3%, *P* < .001), whereas other vital parameters showed no significant differences between the groups. Leukocyte counts (10.9/nL ± 7.6 vs 9.4/nL ± 4.2, *P* < .05) and C-reactive protein (8.8 mg/dL ± 10.1 vs 4.4 mg/dL ± 5.8, *P* < .001) were significantly higher in the inpatient group than in the outpatient group. In contrast, procalcitonin levels were not significantly different between groups.

As a diagnostic consequence, inpatients received significantly more computed tomography scans (34/70 vs 3/65, *P* < .001) than outpatients. Pulmonary infiltration was more frequently detected in the inpatient group (39/70 vs 5/65, *P* < .001).

Multivariate analysis showed that malaise (OR 8.390, CI 1.387–50.731, *P* = .021) and detection of pulmonary infiltrations (OR 12.563, CI 3.613–43.680, *P* < .001) were the only independent predictors of inpatient treatment (Table 2).

**Table 2:** Multivariate logistic regression analysis for endpoint of RSV + and inpatient admission, Nagelkerke *R*^2^ = 0.475.

Category	Variable	Regression	Standard error	*P*-value	Odds ratio (95% CI)
Patient characteristics					
	Age > 60 years	−0.189	0.647	.770	0.828 (0.233–2.942)
Symptoms					
	Malaise	2.127	0.918	**.021**	8.390 (1.387–50.731)
	Dyspnea	−0.238	0.569	.675	0.788 (0.258–2.403)
	Fever	−0.595	0.607	.327	0.551 (0.168–1.813)
	Headache	−0.230	0.728	.752	0.794 (0.191–3.305)
Vital signs					
	Oxygen saturation < 95%	0.422	0.590	.474	1.525 (0.480–4.842)
Lab results					
	C-reactive protein > 7.0 mg/dL	0.142	0.557	.798	1.153 (0.387–3.432)
Imaging					
	Pulmonary infiltrate	2.531	0.636	**<.001**	12.563 (3.613–43.680)
Preexisting conditions					
	Pulmonary	0.269	0.553	.627	1.308 (0.443–3.866)
	Tumor	0.279	0.544	.609	1.321 (0.455–3.838)
	Immunosuppression	1.083	0.891	.224	2.954 (0.515–16.934)
	Hypertension	1.039	0.604	.086	2.825 (0.865–9.231)
	Constant	−2.708			

*P* < 0.05.

## 4. Discussion

The results of this study confirmed that RSV is a relevant pathogen among adult patients presenting to the ED. The virus can cause a severe course of the disease with relevant mortality, especially in the context of serious medical conditions.

Although the clinical picture seems similar to many common respiratory viruses, with malaise, cough, and dyspnea being the most common complaints in our cohort, the clinical course may even be more serious in this setting, with an inpatient rate of more than 50% in our group of ED patients.

### 4.1. General considerations

**Figure 3.**
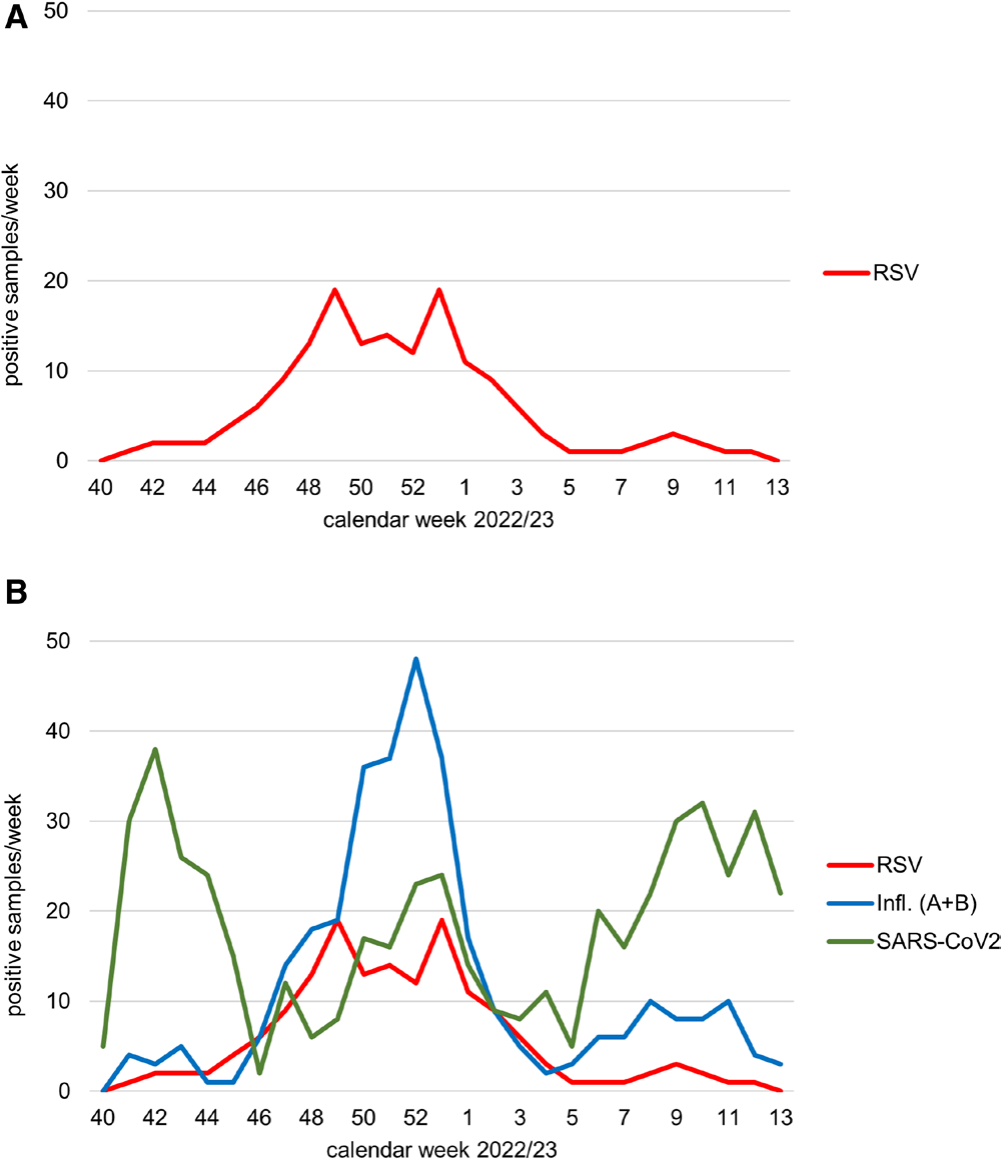
(a) Time course of RSV. (b) Time course of RSV, influenza, and SARS-CoV-2.

The RSV winter season 2022 had an early peak in December, at the same time as the influenza peaked (Fig. 3a and b). Usually, the RSV peak is found later in January and February in the Northern Hemisphere.^[2]^ However, coinfections with both viruses are rarely detected. SARS-CoV-2 showed no significant peaks during the winter term, but was continuously present. Of the 157 RSV+ patients, only 10.8% (17/157) were asymptomatic according to the predefined symptoms of respiratory viral disease and were therefore excluded. However, the rate of asymptomatic courses seems higher in the general population, with a reported prevalence of 42% for RSV and 33 to 41% for SARS-CoV-2.^[12–14]^

Although the prevalence of RSV was lower than that of SARS-CoV-2 and influenza (2.0% vs 6.1% vs 4.0%, respectively) in our cohort, the severity of disease might even be higher with this pathogen, as confirmed by other groups.^[15]^

### 4.2. Patients

With an inpatient rate of nearly 50%, RSV is a relevant pathogen among patients presenting to the emergency department. However, many mildly symptomatic patients will not even contact the healthcare system or will be symptomatically treated by their family doctor. Although 50% of the adult RSV+ patients in our ED could be discharged after a thorough clinical evaluation, the same number required inpatient treatment, with a mean duration of stay of 9.4 days. Other groups reported that among elderly inpatients, RSV results in a longer duration of stay than influenza infection.^[16]^

In our study, the inpatient group was significantly older than the outpatient group with a significantly higher rate of relevant comorbidities, such as pulmonary diseases, tumors, and immunosuppression. Several other groups confirmed the more severe course of RSV in these specific patient groups with comorbidities.^[7,8,17]^

The inpatient group reported significantly more frequent malaise, dyspnea, and fever, whereas headache was significantly more prevalent among outpatients. Among a cohort of coronavirus disease 2019 (Covid-19) patients from the same center, we also demonstrated a significantly higher prevalence of dyspnea in the inpatient group and significantly more headaches in the outpatient group.^[18]^ Dyspnea might therefore be a warning sign of lower respiratory tract involvement and a more severe course of disease in respiratory viral infections.

Our inpatient group showed significantly lower oxygen saturation in ambient air and higher levels of C-reactive protein, as well as a higher leukocyte count. Considering the higher rate of dyspnea and computed tomography scans among prospective inpatients with pulmonary infiltrates (56% of cases), it is very likely that involvement of the lower respiratory tract is crucial for the more severe course of disease in this group. The high rate of pulmonary infiltrates among inpatients, along with a significantly higher level of C-reactive protein, suggests bacterial superinfection/pneumonia as a possible mechanism for the more severe course of the disease. This hypothesis is supported by a study of 607 elderly patients with RSV, where lower respiratory tract involvement and bacterial superinfection were diagnosed in 71.9% and 12.5% of the cases, respectively.^[6]^ In contrast, the rate of lower respiratory tract involvement during RSV infection in former healthy adults is lower, with only 26% of cases.^[19]^

Nearly one-third (21/70) of our inpatients required intermediate care/ICU treatment. The mortality rate of 9% in our inpatient group reflects the findings of epidemiological research, with mortality rates of 6 to 8% among hospitalized RSV patients older than 60 years.^[20]^ A recent German study covering the same study period reported an ICU admission rate of 30% and a mortality rate of 11% among adult RSV+ inpatients.^[15]^

Whereas comorbidities and the abovementioned clinical signs were significantly more frequent in the inpatient group, presence of pulmonary infiltrations and malaise were the only independent predictors of inpatient treatment in the multivariate analysis. Both conditions reflect the poor clinical condition of RSV patients with lower respiratory tract involvement and pneumonia. Patient age and comorbidities, pulmonary diseases, tumors, and immunosuppression were not independent predictors of hospitalization, possibly because of a correlation between increasing age and comorbidities.

Interestingly, the cycle threshold value of the PCR test, a surrogate parameter of viral load, was not different between outpatients and outpatients in this study. In contrast, other studies have demonstrated that a higher nasal viral load may be an independent predictor of respiratory failure.^[10]^ However, it must be noted that comparisons between cycle threshold value or viral copy numbers are difficult because prodromal versus convalescent infections, which strongly influence these parameters, can often not be distinguished in an ED setting.

## 5. Conclusion

Our data demonstrate that older patients and those with relevant preconditions, such as pulmonary diseases or immunosuppression, carry a higher risk for a severe course of RSV infection. In this group, a thorough clinical evaluation should be performed, with a focus on lower respiratory tract involvement. Once lower respiratory tract involvement is present, as suggested by pulmonary infiltration, inpatient treatment is often required. RSV vaccination might be a future strategy to reduce morbidity and mortality in this vulnerable group.

## 6. Limitations

All data were collected retrospectively from medical records; therefore, data entry errors cannot be completely excluded. The study was monocentric with a limited number of patients and should therefore not be generalized. Furthermore, the emergency department of a tertiary care/university hospital might select a patient group with more severe preconditions, such as tumors or immunosuppression.

Although all included patients were symptomatic according to predefined symptoms, the primary reason for presentation remains unclear in some cases as well as the specific origin of their complaints.

Pulmonary imaging was performed at the discretion of the emergency physician; therefore, the rate of pulmonary infiltration may be underestimated in some cases.

## Acknowledgments

We thank all the emergency department employees for their support during difficult times. We thank Mr. Henrik Braitsch for his excellent support in extracting patient data from the medical records.

## Author contributions

**Conceptualization:** Christian M. Kramer, Carina Elsner, Ulf Dittmer, Christian Taube, Clemens Kill, Joachim Risse.

**Data curation:** David Fistera, Christian M. Kramer, Randi Katrin Manegold, Joachim Risse.

**Formal analysis:** David Fistera, Christian M. Kramer, Joachim Risse.

**Investigation:** Carina Elsner, Ulf Dittmer.

**Methodology:** David Fistera, Christian M. Kramer, Carina Elsner, Ulf Dittmer, Christian Taube, Clemens Kill.

**Software:** Joachim Risse.

**Supervision:** Ulf Dittmer, Clemens Kill.

**Validation:** Carina Elsner, Christian Taube, Joachim Risse.

**Writing – original draft:** David Fistera, Christian M. Kramer.

**Writing – review & editing:** David Fistera, Randi Katrin Manegold, Carina Elsner, Ulf Dittmer, Christian Taube, Clemens Kill, Joachim Risse.

## Supplementary Material


